# Vulnerable persons in society: an insider’s perspective

**DOI:** 10.1080/17482631.2020.1863598

**Published:** 2020-12-28

**Authors:** Wilma Numans, Tine Van Regenmortel, René Schalk, Juliette Boog

**Keywords:** Societal vulnerability, vulnerable persons, social inclusion, social work, self-reliance, social participation

## Abstract

**Purpose**: Self-reliance and social participation are strongly promoted by social policy. Both concepts are linked to the concept of vulnerability, for people who do not meet these standards are labelled “vulnerable people”. In this paper, the insider’s perspective takes central stage by seeking to explore what it means to be labelled a “vulnerable person”, and through this to further our insight into the meaning of the concept of vulnerability.

**Method**: Thirty-three in-depth interviews were conducted with 16 allegedly vulnerable people. The data were subjected to thematic content analysis.

**Results**: Our analysis revealed three main dimensions and eight sub-dimensions of perceived vulnerability, outlining an insider’s concept of vulnerability. This concept includes manifestations of vulnerability, feelings coexisting with vulnerability, and the image of vulnerable people.

**Conclusion**: The perception of vulnerability changes when interacting with others in society, especially with social policy implementers. In this interaction, the perceived vulnerability increases and becomes societal vulnerability. It concerns a dependency situation in which one’s strength and self-determination are not recognized, and the help needed is not provided. By acknowledging the insider’s perspective, social policy can fulfil a more empowering role towards “vulnerable people” and contribute to people’s well-being.

## Introduction

Self-reliance and social participation are top priority issues in the policy of the Netherlands. This is in line with the policy of the European Union (EU), of which the Netherlands is a member state. The EU strategy is to contribute to the achievement of smart, sustainable, and inclusive growth (Gros & Roth, 2012), which implies that an inclusive society will enable both economic welfare and personal well-being (Rutenfrans-Stupar, 2019). Although self-reliance and social participation enjoy priority within the policy of the EU, both themes are strongly emphasized in Dutch policy. This started with the King’s speech in 2013 (Rijksoverheid, 2013), concluding that the social security system underlying the traditional welfare state would eventually become financially untenable (Bruggeman et al., 2018; Rijksoverheid, 2013). Since then the Dutch government has worked towards transforming the traditional welfare state into a so-called “participation society” (Rijksoverheid, 2013). Costs needed to be saved and reduced.

To save costs a system change was needed. This system change was realized in 2015 through the decentralization of legislation and the implementation of new legislation, such as the Social Support Act 2015 and the Participation Act 2015 (Bruggeman et al., 2018; Vereniging van Nederlandse Gemeenten, 2013).

To reduce costs, the objective was to bring about a change in mentality among citizens. Citizens’ appeal to government support should decrease. To achieve this change in mentality, self-reliance and social participation are being highly promoted. This implies that citizens take responsibility for their own life, take care of themselves and each other, support each other, and play an active role in society, preferably by doing paid work. Citizens should rely less on government support. With the so-called leading principle of “own strength” the Dutch government wanted to clarify that an appeal for government support should not be an automatism (Bredewold et al., 2018; Bruggeman et al., 2018). Only when there are no other resources at hand, such as care by family members or money, an appeal to government aid becomes an option (Bruggeman et al., 2018; Rijksoverheid, 2013; Van Houten et al., 2008). Social security and professional care are reduced.

Self-reliance and social participation are expected of all citizens. It is the social norm that everyone should meet. Citizens who are not or insufficiently self-reliant or able to participate—who do not meet the standard—are labelled “vulnerable people” in Dutch society (Eugster et al., 2011, 2017; Putters, 2018; Winsemius, 2011).

Although literature study has shown that there is no clear definition of vulnerability or vulnerable people, common denominators can be found in the literature about vulnerability and/or vulnerable populations (Eugster et al., 2011, 2017; Van Regenmortel, 2008; Winsemius, 2011). Usually it concerns people who do not enjoy full physical, psychological and social well-being (Bruggeman et al., 2018; Jehoel-Gijsbers, 2004; Metz, 2009; Provinciale Raad voor de Volksgezondheid en Maatschappelijke Zorg in Noord-Brabant [PRVMZ], 2010), and as a result are (at risk of) falling behind in society or become socially isolated (Eugster et al., 2017; Movisie, 2013; Schuyt, 2000; Van Regenmortel, 2008; Winsemius, 2011; Wolf, 2019).

In addition, other common characteristics mentioned in literature are: (1) accumulation of problems or limitations (multi-complex problems); (2) feelings of powerlessness and distrust; (3) disrupted communication; (4) limited or no access to resources; (5) marginality; (6) imbalance in burden and capacity; (7) dependency situation; (8) low self-esteem (Eugster et al., 2011; Sociaal en Cultureel Planbureau, 2014; Van Regenmortel, 2009, 2008; Winsemius, 2011).

Overlooking these denominators and characteristics, it concerns people who do not or insufficiently meet the social standard,—of self-reliance and social participation -, and are therefore included in the category of “vulnerable people”. As such “vulnerable people” and “vulnerability” can be understood as a concept, based on an outsider’s perspective; the perspective of non-vulnerable people. This concept allows outsiders to label people as “vulnerable” and relegate people to the group of vulnerable people.

The question is, if you are included in the group of vulnerable people and classified as a “vulnerable person”, how do you perceive this? What does it mean to allegedly be vulnerable? Based on our literature study we found that neither in Dutch social policy nor in the literature the perceptions of allegedly vulnerable people themselves are included. Social policy and theory usually deal *about* people from vulnerable populations, rather than *with* these people (Abma et al., 2011, 2009; Siesling & Garretsen, 2014; Van Regenmortel et al., 2013). The insider’s perspective on the concept of vulnerability is lacking. Labelling people “vulnerable”, after all, does not automatically lead to an in-depth understanding of what it really means to be labelled “vulnerable” for the people concerned.

This insider’s perspective is important because due to reduced social security and professional care, the number of persons labelled “vulnerable” is growing (Bijl et al., 2015, 2017; Centraal Bureau voor de Statistiek, 2019; Coalitie Erbij, 2015; Putters, 2018; Sociaal & Cultureel Planbureau/Centraal Bureau voor de Statistiek, 2014; Wolf, 2019). Policies aimed at realizing an inclusive society, enabling economic welfare and personal well-being for all citizens, seem to achieve the opposite: social exclusion. The perceptions of “vulnerable people” themselves can provide new insights which can be included in social policy and the existing concept of “vulnerability”, and thereby contribute to an inclusive society.

In this paper, we present our findings based on an in-depth bottom-up approach to explore the concept of vulnerability from the perception of allegedly vulnerable people themselves: how do they perceive being labelled “vulnerable”?

With this, we aim to further our insight into the meaning of the concept of vulnerability by comparing the perceptions of allegedly vulnerable people regarding this concept—their subjective and experienced reality expressed in words—with the current outsiders’ perspectives of social policy and theory on vulnerability. We explore to what extent these different perspectives meet or differ from each other, striving to contribute to a more complete concept of vulnerability which includes the perceptions of allegedly vulnerable people.

## Methods

### Design

The empirical data presented in this paper stem from a study (2017–2019) in which the perception of allegedly vulnerable people on vulnerability takes central place. The study was conducted in a medium-sized city in the Netherlands (217.259 inhabitants) as part of a PhD research project that started in 2015.

A qualitative method was used, as qualitative methods help researchers to gain a deeper understanding of the research topic (Sutton & Austin, 2015). Moreover, qualitative methods are well-suited to identify processes and patterns which lead to better accounts of the experiences of respondents, and to give voice to those who are otherwise silenced (in this study allegedly vulnerable persons) (Janssen et al., 2011; Ungar, 2003).

This study used a naturalistic inquiry, which aims to understand the particularities of a phenomenon in its natural setting and based on the perception of those involved (Lincoln & Guba, 1985).

### Data collection

Clarifying the perception of respondents requires a conscious and linguistic construction of meaning. Therefore, a dialogue between researcher and respondent is needed (Baarda et al., 2005; Tromp, 2004). We chose in favour of in-depth interviews. In-depth interviews provide space for respondents’ narratives and allow for searching specific experiences and feelings of respondents which are important to the perception of vulnerability. Narratives are subjective and reveal what the narrator finds important and wants to reveal. They depict situations from the narrator’s perspective (Abma & Widdershoven, 2005).

The interviews were prepared and conducted by a research team, consisting of the first author (principal researcher) and eight co-researchers, four persons from vulnerable populations and four professional social workers; all of them being more or less familiar with an insider’s perspective on, and experiential and/or practice knowledge of vulnerability.

The research team was assisted by an external researcher—an anthropologist with extensive experience in conducting qualitative (grounded) research. The external researcher started as a member of the sounding board group in the research project and during the research project became a “critical friend” (mentor) for the principal researcher and co-researchers (Van Regenmortel et al., 2013, 2016).

All co-researchers wanted to conduct interviews with persons from vulnerable populations and did under the supervision of the first and fourth author. To conduct the in-depth interviews we used the Interview Guide Approach; a widely used format for qualitative interviewing. In this Approach, the interviewer has a previously specified outline of topics or issues to be covered, but is free in deciding the sequence and phrasing of questions during the interview (Patton, 1987). To ensure reliability and validity, all co-researchers were well prepared in how to conduct in-depth interviews through theoretical and practical (interview) training and reflexive sessions, concerning for example, extensive discussion about the topic-list and terms used during the interviews, workshops including role-playing, and by regular feedback sessions during the data collection period based on recorded interviews, where the purpose of the interviews repeatedly was highlighted.

The in-depth interviews consisted of two interviews with each respondent. Interview 1 focused on the theme “Me & vulnerability”. The first interview started with becoming acquainted with the respondent, followed by an exploration of what vulnerability means to the respondent, how it manifests itself, how vulnerability is perceived, and in which life domains the respondent experiences vulnerability. This in order to gain a good understanding of the perceived vulnerability at the individual (personal) level.

In interview 2 the theme “Others and process” was central, focusing on the actors and factors that play a role in perceiving vulnerability, also over time. The second interview started with a summary of the content of the first interview and a check of its correctness (member check). Subsequently, the respondent’s social life was discussed: which persons and what elements play a role in the origin, continuation, aggravation and reduction of the perceived vulnerability? When and how? And how does the respondent himself or herself deal with perceived vulnerability? In addition to the individual level, interview 2 also covered the interactional level and aimed at gaining insight in possibilities for improvement to reduce perceived vulnerability.

In total 33 interviews were conducted: 2 interviews with 13 respondents (interview 1 & 2), 3 interviews with 2 respondents (interview 1 & 2), and 1 interview with 1 respondent (interview 1). Deviations from the standard 2 interviews procedure were made with the consent of the respondent.

### Respondents

As we selected a qualitative explorative research approach, the sample size was limited to 16 respondents and based on purposeful sampling (Marshall, 1996; Smaling, 2014, with reference to Patton, 1990, pp. 182–183; 2002, pp. 243–244). Qualitative research does not numerically reflect the total population, but aims to gain insight into and an understanding of complex psychosocial phenomena (Marshall, 1996). After interviews with 16 respondents saturation was reached (Meadows & Morse, 2001).

The following selection criteria were used: (1) eligible respondents meet the current definition (common denominators) and/or characteristics of “vulnerable persons” as indicated in the Introduction of this paper; (2) are at least 23 years old (from the point of view of (assumed) “wisdom of life” and/or life experience and reflective capacity; (3) understand the Dutch language and can express themselves verbally; (4) respondents perceive vulnerability: a personal feeling of vulnerability and/or the perception of feeling vulnerable in a certain domain and/or aspect. Diversity in age distribution and gender were also taken into account. Eligible respondents who were under medical or specialist psychological or psychiatric treatment were excluded from the study, in order to prevent that participation in the study could in any way have a negative impact on the treatment received by the person concerned.

Pre-selection of eligible respondents took place by members of the advisory board group of the research project who work with and/or are in touch with people from vulnerable populations (n = 4). The defined selection criteria were leading in the pre-selection. Eligible respondents who were pre-selected by advisory group members were discussed with the principal researcher to determine if they were suitable for participation. The principal researcher decided the final selection based on the selection criteria, richness of the case, and variation. Based on the final selection by the principal researcher, the respondents selected were recruited by advisory board members through personal contacts with respondents. When recruiting, an information letter was provided to the participant by the advisory board member. In case of consent to participate in the study an interview was planned.

The 16 respondents ranged from 31 to 75 years of age (mean 49), and included 7 men and 9 women. Of the 16 respondents, 8 were respondents who had no complete status of psychological well-being, but suffered from mental illness such as personality disorder, borderline disorder, depression, panic disorder, and hypersensitivity. The other 8 respondents were persons who had no complete status of physical well-being, but suffered for instance, from progressive muscle disease, multiple sclerosis (MS), cerebral palsy (CP), blindness, and heart and lung disease. 3 respondents had an income from employment; the other 13 respondents received social benefits.

The respondents decided where and when the interviews took place. Most of the interviews took place in the respondents’ own homes. The interviews lasted approximately 2 hours (varying between 61 and 178 min).

### Analysis

Our analysis was guided by Thematic Analysis, in which patterns or themes within qualitative data are identified systematically (Braun & Clarke, 2006; Guest et al. (2012); Maguire & Delahunt, 2017). Characteristic for Thematic Analysis is that it is related to both phenomenology and grounded theory (Charmaz, 2006; Guest et al., 2012), two approaches that formed the core of the study.

In our analysis multiple (co)researchers were involved (check-coding): the principal researcher (first author), a co-researcher with a scientific background and familiar with data-analysis, and the external researcher (fourth author). All transcripts were coded by the principal researcher and independently co-coded by the co-researcher and the external researcher.

Our analysis was based on the collected data and these in turn were streamlined by the topic list, aimed at answering the research question. We started our analysis by using a combination of interpretation and open coding, assisted by ATLAS.ti (version 7 for Windows). As a starting point, we discussed some preliminary ideas about the codes and developed some initial codes as point of departure for the coding based on reading some transcripts. We did not have pre-set codes or a pre-existing model or frame in which we tried to fit the data. On the contrary, our analysis was driven by the data itself.

We developed and modified the codes as we worked through the coding process by regularly comparing our codes. In case of inconsistencies, doubt and/or disagreement in co-coding, the coders discussed until consensus was reached about a code. This process led to a final code-tree. Main themes on the code-tree are for instance, vulnerability as experienced by participants, process of vulnerability over time, perception of contacts in relation to vulnerability, and suggestions for improvement to institutions.

The next step in our analysis consisted of moving back and forth between identifying, reviewing and defining themes. This step was executed by the first and fourth authors. The earlier involvement of the co-researcher was discontinued due to lack of time. Coded segments of coherent data under each (sub)code were grouped to discern patterns and define themes.

During this phase, we also went back and forth between our coded empirical data and theory. In order to (re)construct theory and concepts driven by our empirical data, and gain further insight in empirical data, we searched for relevant theories and concepts in literature. The literature functioned as “sensitizing concepts”: not as prescriptions of what to see, but as directions in which to look (Blumer, 1969). We started with an inductive approach and during our analysis we jumped from inductive to deductive and back again. This is in line with Jackson’s and Mazzei’s (2013) approach “thinking with theory”, in which the interaction between empirical data and theory (in our case: sensitizing concepts) takes place. It is precisely this interaction that can lead to further steps and insights in both directions, allowing surprising knowledge to be produced (Bos, 2016). Helpful in going back and forth between data and sensitizing concepts was the reflexivity journal that we created and maintained from the outset. In this journal, we documented our steps, our (early) impressions, and reflections on potential findings. This was useful for reflecting on emergent patterns, themes and concepts (Saldana, 2009).

At various times in this stage, we briefed the co-researchers on preliminary interpretations and findings as a form of member check. At one time we performed a member check with the advisory board group. All input was taken into account and, when possible, also processed. This is in line with Lincoln and Guba (1985) who consider member checking as a process that occurs continuously during the research project, and comprises the testing of data, analytic categories, interpretations and conclusions with members of the stakeholder group(s). It contributes to the credibility and reliability of the researchers’ work. In addition, it is a “strong beachhead toward convincing readers and critics of the authenticity of the work” (Lincoln & Guba, 1985, p. 315).

### Ethics

All interviews were recorded and transcribed verbatim with respondents’ permission. All persons who participated in the study gave written informed consent for each interview. In providing consent, respondents were given the option to withdraw their consent at any time. This did not occur. The confidentiality of the respondents was ensured by replacing the respondents’ names with codes. Only the principal researcher (first author) has access to these codes. Respondents received a compensation of € 20 for participating in the study. The research protocol was approved by the Ethics Review Board of Tilburg University (EC-2017.35t).

## Findings

In our findings section, we present how vulnerability and being labelled a “vulnerable person” are perceived by our respondents. Starting point is the subjective experience of respondents, outlining the respondents’ perceptions on vulnerability. In our discussion we reflect on our findings and compare the respondents’ perceived vulnerability with the concept of vulnerability as described in literature. In addition, we discuss the social political concepts of “social participation” and “self-reliance”. The results of this comparison can contribute to the further completion of the concept of vulnerability.

The cited quotes of respondents are translated from Dutch by the first author.

### Perceptions on vulnerability: an insider’s perspective

When exploring the respondents’ perceived vulnerability we found three main dimensions in the data that reflect their perception. Our data also revealed sub-dimensions within each main dimension. First, the main dimension *manifestations of vulnerability*, understood as how vulnerability manifests itself and what it means for everyday life. Sub-dimensions found in the data contained (1) type of vulnerability as expressed by the respondents in mental illness or physical defects, (2) dealing with manifestations of vulnerability (behaviour), and (3) limitations in social participation.

Second, the main dimension *feelings coexisting with vulnerability*, understood as the way in which vulnerability in itself—the illness or defect—is experienced by respondents, and how vulnerability is experienced in interaction with others in society. Subsequent sub-dimensions found in the data were (1) feelings at the individual level and (2) feelings at the interactional level.

Third, the main dimension *image of vulnerable people*. Here sub-dimensions that were revealed in the data include the way in which respondents think (1) about themselves (self-image), (2) about other people from vulnerable populations, and (3) how respondents think that others in society—non-vulnerable people—see them.

We will describe our findings, using these main dimensions and present the sub-dimensions within each of the dimensions. In the discussion, we end our findings with a reflection on these dimensions. Our key findings are summarized in Table I.

**Table I.** Key findings perceived vulnerability summarized

Perceived vulnerability: an insiders perspective		
Dimension:	Sub-dimension:	Components:
Manifestations of vulnerability	Type of vulnerability	Mental (psychological) vulnerability
		Physical vulnerability
		Financial vulnerability
	Dealing with manifestations of vulnerability (behaviour)	Negative, e.g., silencing, withdrawing, going beyond limits
		Positive, e.g., planning and making informed choices; adapting
	Social participation	Limitations in social participation: reduced participation, curtailment of the social network due to illness/disorder
		Contacts/social network
		Participation in life domains: family life, leisure activity, volunteer work, education, paid work, and institutional life
Feelings coexisting with vulnerability	Feelings at the individual level	Fear, anxiety, shame, loneliness, insecurity, anger, and feelings of emptiness, brokenness, and heaviness
	Feelings at the interactional level	Inferior in society, dependency and not having self-determination, powerlessness, frustration, being misunderstood/lack of empathy, disappointment, being patronized, and being stereotyped and stigmatized
Image of vulnerable people	Self-image	Expressed in personal competencies (positive and negative)
	Image of other allegedly vulnerable people	Expressed in personal competencies (negative)
	Perceived image of society on “vulnerable people”	Expressed in stigmas (negative)

#### Manifestations of vulnerability

##### Type of vulnerability

Respondents expressed the type of vulnerability in mental illness and physical defects, for example, personality disorder, borderline disorder, depression, panic disorder, hypersensitivity, progressive muscle disease, multiple sclerosis (MS), cerebral palsy (CP), blindness, and heart and lung disease. Respondents also mentioned financial deficits or problems. Translating this to the type of vulnerability we found three main types of vulnerability: mental (psychological) vulnerability, physical vulnerability, and financial vulnerability. Respondents also expressed that these types of vulnerability do not exist separately from each other, but often in combination. All respondents who mentioned mental illness as a cause of experienced vulnerability also suffer from physical disorders, for example, fatigue, muscle strain, and dizziness. Some of these respondents also have to deal with financial deficits as a consequence of their illness and disorder, for instance, through not being able to work. Some respondents who mentioned physical defects as a cause of experienced vulnerability also mentioned psychological issues and financial deficits. However, the combination of experienced physical vulnerability and (leading to) mental vulnerability seems less self-evident than vice versa.

##### Dealing with manifestations of vulnerability (behaviour)

With this sub-dimension, we refer to the way respondents react to or deal with their mental illness and physical defects or shortcomings. This type of behaviour is directly linked to these manifestations. Illustrations of expressions of dealing with manifestations of vulnerability in daily life: not indicating or discussing that you feel bad, planning your daily life and making informed choices about what you are able or not able to do, such as withdrawing from social life, not going beyond your limits, and adapting to the situation by asking for help, using new (care) technologies for people with disabilities, and/or using (specific) medical devices such as a walker, wheelchair, mobility scooter, adapted bicycle, and orthopaedic shoes.

##### Limitations in social participation

The majority of the respondents mentioned both reduced participation in social activities such as forest walks, outings, visits, and curtailment of their social network (including education and paid work), resulting from the illness, defects and/or shortcomings. On the other hand, reflecting on the theme “contacts” and “daily activities”, all respondents indicated that they have social contacts, interact with others in society, and participate in various life domains. Contacts include family members, friends, neighbours, participants in organized social activities and/or (voluntary) support groups, colleagues in volunteer work, sometimes fellow students, in a few cases work colleagues, and contacts with professionals from service and care delivery organizations. Subsequently, life domains retrieved from the data concern family life, leisure activity, volunteer work, education, paid work, and institutional life. Most of the respondents related that their contacts are structural and that they vary from telephone contact, personal contact, social media contact and/or written contact (in case of contact with professionals from service and care delivery organizations). None of the respondents indicated that they did not participate socially. On the contrary, most of the respondents expressed that they are active in volunteer work—some of the respondents in multiple forms of volunteer work—and leisure activity such as sports, shopping, acting (theatre group), and playing music.

#### Feelings coexisting with vulnerability

##### Feelings at the individual level

Respondents’ feelings at the individual (psychological) level concerns how it feels to be vulnerable; to have an illness, defect or shortcoming? All respondents expressed negative feelings at the individual level. Although the data revealed a wide spectrum of feelings expressed by respondents’, we found common denominators such as fear, anxiety, shame, loneliness, insecurity, anger, and feelings of emptiness, brokenness, and heaviness. In the wide spectrum of feelings expressed the data also revealed that these common denominators are nuanced differently by the respondents. For example, some of the respondents described the denominator “fear” as fear of relapse (of homelessness and depression), while other respondents described “fear” as fear of the continuing physical decline and the resistance felt to accepting the use of medical devices such as a wheelchair. Other respondents linked “fear” with “insecurity”. For example, feelings of fear and insecurity about not having diplomas and work, or feelings of fear and insecurity about the availability of care and a disabled toilet when going out. Another example is the denominator “anger”. One respondent described this as follows: “I am very angry about the things I have not done, like having children and a job. I am upset by the diagnosis and help coming too late” (respondent 16, female, 46). In the expressions of other respondents the denominator “anger” was accompanied by feelings of frustration and discouragement. One respondent described this as follows: “I feel anger and I can’t blame anyone. That is frustrating. The disease is an obstacle to the life I wished for” (respondent 21, female, 35). Another respondent: “I feel discouraged. Due to my illness, there is no solution. If there is a solution for vulnerability then you are not vulnerable, for example, in the case of homelessness. But illness, it happens to you, because there is no solution” (respondent 22, female, 65). And another respondent: “I am trapped in my body. Physically I can’t do anything anymore and I don’t see any improvement. Out of frustration I sometimes throw things around” (respondent 6, female, 40).

The data also revealed differences in expressed feelings between respondents who mentioned mental illness as a cause of experienced vulnerability and respondents who mentioned physical defects as a cause of experienced vulnerability. In the first group a number of respondents expressed feelings of inferiority in society and a lack of self-esteem due to their mental illness. Some of the respondents also mentioned feelings of deep and old pain. In contrast, these feelings were not addressed by respondents whose experienced vulnerability is caused by physical defects. In this group a majority of the respondents expressed sadness and feelings of physical deterioration and loss of function and grip due to physical disorders. For example, “It is a process of surrendering, from walking to rolling [in a wheelchair], of always needing help instead of sometimes” (respondent 21, female, 35). Another respondent expressed this as follows: “It feels like slowly sliding down the tiled roof, stepping into the abyss” (respondent 18, male, 60). Some of these respondents compared these feelings to a grieving process, while others mentioned feelings of dependence: dependence on others, transport, medical devices, services and care to be able to live life with physical disorders.

Remarkably, besides negative feelings, a few respondents also expressed positive feelings at the individual level. This concerns the feeling of victory when something succeeds, and the feeling of satisfaction. For example, “Despite my condition I live a fairly complete life, I live like a normal person: I am married, I have children and I am divorced” (respondent 9, male, 55).

##### Feelings at the interactional level

Respondents’ feelings at the interactional level concern how their vulnerability (vulnerability in itself) feels in interaction with others in society.

All respondents expressed that their vulnerability felt solely negative at the interactional level. Their feelings concerning vulnerability differ in comparison with the feelings at the individual level. In the respondents’ expressions, we found the following common denominators: feelings of being inferior in society, of dependency and not having self-determination, of powerlessness, of frustration, of being misunderstood, of disappointment, and of being patronized. In line with the individual level the expressed feelings by the respondents often were interrelated.

The main common denominator addressed by the respondents is the feeling of being inferior in society and not taken seriously. For example, “I feel written off by society. They don’t expect anything from me anymore because I’m in a wheelchair. You are taken for a fool when you are in a wheelchair” (respondent 18, male, 60). Or another example: “I am not seen as a person, but as a poser or an alcoholic” (respondent 16, female, 46). Some of the respondents connected the feeling of being inferior and not taken for full of the feeling of being stigmatized. As one respondent quoted: “A disabled person will be ill a lot” (respondent 9, male, 55). Or: “I cost a lot, I’m unprofitable” (respondent 21, female, 35).

The main common denominator is followed by a feeling of dependency and not having self-determination. In the expressions of a majority of the respondents who mentioned physical defects as a cause of experienced vulnerability, this denominator was accompanied by feelings of powerlessness. For example regarding social benefits, help and medical devices: “Your fate is in their hands. You are not in charge. You depend on the other person who can play God” (respondent 17, female, 34). The data also revealed that the expressed feelings of powerlessness and not having self-determination are linked to the feeling of being patronized. For example, “Caregivers who treat you like a baby ” (respondent 21, female, 35).

Some of the respondents who mentioned mental illness as a cause of experienced vulnerability also expressed feelings of powerlessness, often accompanied by feelings of despondency, injustice and frustration due to a lack of adequate psychological and social assistance. For example, “It is a scar, but you can pull the string out with tweezers. The wound opens. There is a little pus, but it is not enough to heal the real wound. In the wound lies a request for help, but it is not treated, it is not really addressed. I am snowed under in an avalanche of what is important to the caregiver” (respondent 5, male, 62).

Feelings of frustration were also mentioned by some of the respondents who deal with physical disorders, caused by the difficulty of getting the necessary social benefits, adequate care and medical devices. For example, “I feel frustrated because I constantly have to prove that I need medical devices because of my illness. I get tired of fighting for my rights” (respondent 6, female, 40). Although the form of the needed help differs, respondents from both groups expressed feelings of frustration because the help (immaterial and material) does not arrive or does not arrive on time.

Another common denominator retrieved from the data concerns the feeling that the vulnerability is misunderstood. According to a number of respondents this feeling is caused by a lack of empathy. For example, chronic fatigue which is compared by others to “I also get tired sometimes”. Or a blind respondent being dropped off by a taxi, but not escorted to the front door. In the expressions of respondents who mentioned mental illness as a cause of experienced vulnerability, the feeling of being misunderstood was accompanied by a feeling of disappointment.

Some of the respondents who suffer physical disorders also expressed a feeling of disappointment. In their experience this is due to a lack of adequate help, others who do not see the respondents’ struggle, and promises that don’t come true. For example, “They promised physical improvement, but it does not happen” (respondent 6, female, 40).

On the other hand, despite these negative feelings of for instance, being misunderstood and lack of empathy, some of the respondents expressed their understanding for being misunderstood by other, non-vulnerable people, reminding themselves of the time when they had no illness or defects. They expressed that before their illness or defect, they also found it difficult to empathize with what it really means to have and deal with an illness or defect in daily life.

#### Image of vulnerable people

##### Self-image

Self-image was indicated in terms of positive or negative personal competencies described by the respondents (addressed in terms of “I am” or “I have”). All respondents expressed perceptions of a positive self-image. Illustrations of “I am” are: “an expert by experience”, “very helpful”, “a world citizen”, “powerful”, “proud and satisfied with what I have achieved”, “an ordinary person in society, not my disease.” Illustrations of “I have” are: “I have a sense of humour”, “a fairly high level of intelligence”, “self-confidence and self-esteem”, “endurance and angelic patience”, “nerve and guts to get things done”, “knowledge of complicated regulations”.

Although all respondents indicated a positive self-image, a few respondents also expressed perceptions of having less positive personal characteristics. For example, “I am insecure and struggle with my self-confidence”, and “I have a lack of self-confidence to present myself”.

##### Image of other allegedly vulnerable people

Respondents reflected also on their perception of other people from vulnerable populations. In line with self-image, respondents’ image of other “vulnerable people” was also expressed in terms of positive or negative personal competencies described by the respondents. A majority of respondents expressed a less positive image of other allegedly vulnerable people than their perceived self-image. Respondents mentioned for example, that other allegedly vulnerable people are lonely and isolated, not good at articulating the help they need, not active in society, not good at overcoming barriers, less articulated and unable to stand their ground. Respondents also mentioned shortcomings in terms of “lack”: allegedly vulnerable people have a lack of tools and self-knowledge, of making the right choices, of energy, of insight into complicated regulations and laws, of expressing their vulnerability, of grip on life, of money, and of the ability to enter into social contacts.

##### Perceived image of society on “vulnerable people”

Regarding the perceived image that others in society—non-vulnerable people—have of allegedly vulnerable people, none of the respondents expressed perceptions of a positive image. On the contrary, the data revealed exclusively negative expressions of respondents indicating stigmatizations. In respondents’ expressions of the perceived image that others in society have of allegedly vulnerable people, we found the following common denominators: vulnerable people are seen as a) inferior (can do nothing, are sick and weak), b) a target group, c) stupid, d) expensive (you cost society money, but you do not yield anything economically), e) dangerous, and f) crazy.

Examples of expressions of these common denominators given by the respondents concerning “inferiority” are: “We do not participate in the participation society because of our physical disabilities. We are weak” (respondent 9, male, 55). “As a Wajonger [type of social benefit] you can’t do anything. Especially don’t try anything, because you won’t achieve anything. You are not complete” (respondent 19, male, 34). Concerning “target group”: “You are a certain target group instead of a person” (respondent 5, male, 62), and “You are manoeuvred into a box. You have to belong to a club that you do not feel like belonging to” (respondent 2, female, 51). Concerning the common denominator “stupid”: “Wheelchair users are physically and mentally disabled. People talk louder. They see us as stupid” (respondent 17, female, 34). Concerning “expensive”: “People with disabilities are too expensive, they cost too much” (respondent 6, female, 40). Concerning “dangerous”: “Psychologically disturbed people are dangerous” (respondent 10, female, 45). And at last, concerning “crazy”: “Vulnerable citizens who are hospitalized, go to the madhouse. Mentally vulnerable people are crazy” (respondent 1, female, 31).

## Discussion

In this paper, we presented the perceived vulnerability as expressed by the so-called “vulnerable persons”. The data collected were disentangled and reveal a layered concept of vulnerability as perceived by the respondents themselves. In looking for commonalities, three main dimensions, each with two or three sub-dimensions of perceived vulnerability were identified (see Table I). Our findings indicate a strong intertwinement between the main dimensions and sub-dimensions allowing the perceived concept of vulnerability to be pictured as a complex whole, consisting of various gearwheels that interact with and influence each other. Noteworthy here is that the influence sometimes can be expected and sometimes is surprisingly unexpected. This again supports the complex reality of vulnerability and allegedly vulnerable persons.

The main characteristic of persons labelled as “vulnerable” manifest themselves in mental illness or physical defects—sometimes together, and in combination with financial deficits—which impact respondents’ handling of vulnerability in their daily lives. Respondents’ daily confrontation with vulnerability affects their social participation as well as their ability to build and keep up social networks. Nevertheless, all respondents accounted for participation in several life domains and they all mentioned a small or wider range of social contacts. Surprisingly this seems to contradict with respondents’ negative feelings found in the sub-dimension of both individual and interactional level, because here we would expect less social activity of our respondents in various life domains. However, in the sub-dimension “dealing with manifestations of vulnerability”, with respect to planning, making informed choices and adapting, respondents showed that they eventually can find their own way in daily life, including social participation—even if felt reduced or limited—as well as in social networking, even if felt curtailed.

The sub-dimension of self-image is in harmony with this. According to respondents, they feel themselves well-endowed with competencies and capabilities. In contrast to the competencies ascribed to themselves, they see exactly these competencies lacking in other allegedly vulnerable people. It seems as if the shortcomings of other vulnerable people are underlined by respondents, because without such competencies it is apparently impossible to maintain oneself in society, to participate socially and be self-reliant.

At this point, it is also worth noting that although respondents feel that they are being stigmatized, they refrain from participating in the social process of stigmatization (Goffman, 1963) and do not stigmatize other allegedly vulnerable people. In this way, by emphasizing competencies the respondents also seem to counterbalance the stigmatization they experience by others—non-vulnerable people—in society. Unlike non-vulnerable people in society, respondents do not write other allegedly vulnerable people off, for competencies can be learned and worked on. At least for the respondents this has worked out well. For it is noticeable that respondents developed and maintained a positive self-image, despite the stigmatization experienced and negative feelings at both individual and interactional level. So, they not only do not write off other allegedly vulnerable people, but not themselves either. Seemingly respondents have the competencies to deal with negative feelings and to be or become resistant to negative imaging and negative experiences with others in society. This can also be understood as a strength in vulnerability, and a process of becoming and perceiving oneself as valuable.

Moreover, by emphasizing competencies, it seems as if respondents see more potential in themselves to contribute to society: they count in society and must be taken serious, despite their limitations and shortcomings due to illness and disease. In short, they accept being vulnerable, but they do not accept being of no value to society, as findings prove that our respondents are socially active in several life domains.

The notion of being socially active leads us to discuss the social political concept of “social participation”. Social policy aims at paid work as the preferred form of social participation, specifically addressed in the Participation Act 2015 (Bruggeman et al., 2018). Measuring tools, such as Arnstein’s (1969) ladder of participation, are used to determine the level of participation, and in this tool paid labour is considered to be the highest form of social participation (Vereniging van Nederlandse Gemeenten, 2010). In this way, a hierarchic order is assumed in which the lower levels of participation are usually valued as inferior, related to lower economic value. However, from an insider’s perspective the lower levels of participation should also be considered as being of value by society. The issue here is not primarily economic value but social value. This implies that the concept of “social participation” has to be nuanced differently: not as a hierarchic order, but as a horizontal order in which all levels of participation are considered equally valuable. Valuable for society as whole, including both non-vulnerable people and vulnerable people. This is inclusivity.

Having reflected on the main and sub-dimensions of the perceived concept of vulnerability and the social political concept of “social participation”, we now confront our findings with the literature describing common characteristics of vulnerable people: (1) accumulation of problems or limitations (multi-complex problems); (2) feelings of powerlessness and distrust; (3) disrupted communication; (4) limited or no access to resources; (5) marginality; (6) imbalance in burden and capacity; (7) dependency situation; (8) low self-esteem (Eugster et al., 2011; Sociaal en Cultureel Planbureau, 2014; Van Regenmortel, 2008, 2009; Winsemius, 2011). When comparing our narratives to these characteristics it is clear that literature has a bleaker view of vulnerable people than our respondents have. Literature gives the impression that vulnerable people suffer all negative aspects and feelings. However, our respondents claim otherwise.

First, some of the characteristics can be diminished, by having and applying adequate competencies. This inherently means that the common characteristics are not fixed facts but can change over time; they are dynamic rather than static. In case of our respondents for example, apparently having social competencies and a (developed) positive self-image can diminish disrupted communication and by this provide access to resources—especially resources in the immediate social environment—which in turn can contribute to a balance in burden and capacity. In this regard literature does not fully meet the reality experienced at the individual level. Competencies play a crucial role in the view of our respondents. This aspect is not taken into consideration in literature as a main characteristic of vulnerable people and we recommend that it be added.

Second, negative aspects (common characteristics) are mainly felt during interaction with others in society. In this respect, at the interactional level, theory and experienced reality agree, at least to some extent. With respect to interaction we discovered three sublevels in the interactional level in respondents’ narratives: (a) the interaction with others from their own social environment; (b) the interaction with others—non-vulnerable people—in society; and (c) the interaction with others who operate in the institutional life domain in which respondents move, such as care professionals and social service providers—the so-called social policy implementers. In line with Janssen et al. (2011) we interpret the third sublevel as the political-societal level, which includes the accessibility of care and help, and the availability of material resources. “Others” at this level are in a sense the gatekeepers to self-reliance and social participation. Their role is to help and support vulnerable people, and contribute to vulnerable people’s well-being. However, according to respondents, the opposite is the case: precisely in the interaction with others at the political-societal level they experience increased vulnerability, consisting of feelings of inferiority, no self-determination, powerlessness, frustration, lack of empathy, disappointment, being patronized, and being stigmatized. This is all the more poignant if, like the respondents, one is factually dependent on others to live one’s life; when more than others one needs help, care and medical devices to be self-reliant and able to participate socially because of one’s (mental or physical) illness or disabilities.

Such negative feelings in interaction with others are not mentioned by respondents when interacting with people in their own social environment, and mentioned less when interacting with non-vulnerable people in society (with the exception of feeling stigmatized). In other words, others at the political-societal level, who would be expected to contribute to reduction of the common characteristics of vulnerable people, seem to achieve the opposite: prolongation of the common characteristics mentioned in literature. For instance, characteristic (3) disrupted communication is only at play at the political-societal level in respondents’ narratives. In addition, the common characteristics also seem to have different meanings in the different interactional levels. For instance: characteristic (7) dependency situation exists both in the interaction with others in respondents’ own environment and in the interaction with social policy implementers. However, dependency on people in their own environment is not perceived as negative, in contrast to the interaction with others at the political-societal level.

The distinction between different interactional sublevels and the felt impact of the type of interaction on vulnerability we derived from an insider’s point of view, is not made in literature concerning common characteristics of vulnerable people, and should be taken into consideration.

However, the role and importance of the interactional level in the experienced vulnerability is mentioned in literature concerning the concept of “societal vulnerability” (Baart & Carbo, 2013; Raad voor Maatschappelijke Ontwikkeling, 2001; Van Regenmortel, 2008; Vettenburg, 1988; Vettenburg & Walgrave, 2002). This concept focuses on the interaction between individual and social structures (social networks and social institutions). According to this concept, vulnerability is not confined to an individual, but vulnerability stems from and reproduces in the interaction between individual and social structures. With interaction at its core, the concept of “societal vulnerability” seems in line with our respondents’ experiences. Vulnerability is mainly a relational fact. According to our respondents, vulnerability is felt differently when interacting with others.

Following our respondents’ perceived vulnerability, the concept of vulnerability from an insider’s perspective can now be described as follows:

Vulnerability is a factual state of mental illness, physical defects and/or (financial) deficits, which makes someone dependent on help from others. If that dependency in the interaction with other people is accompanied by feelings of inferiority in society, of not having self-determination, of powerlessness, of frustration, of being misunderstood (lack of empathy), of disappointment, of being patronized, and of being stigmatized, then we speak of “societal vulnerability”. In short, “societal vulnerability” is an interactional state in which a person is not seen and treated as a full person and in which a person does not get the appropriate help he or she needs and has a right to. Perceived vulnerability increases and it undermines self-reliance and social participation.

The issue of dependency mentioned earlier is crucial for respondents. It increases their perceived vulnerability. This brings us to discuss the social political concept of “self-reliance”.

According to the Dutch government self-reliance includes the ability to carry out the necessary general daily life activities and to run a structured household (Bruggeman et al., 2018). Self-reliance refers to both individual independence—also called “own strength” and “self-care”—as well as the ability to request and receive informal help from people in one’s social environment (Bredewold et al., 2018). To a certain extent, our respondents are self-reliant, be it with help from their own social networks. Nevertheless, this self-reliance is not always sufficient, and external help in the form of government support is needed. This is also a legal right (Bruggeman et al., 2018).

However, according to our respondents, even if this is the case and government aid is necessary and actually something you have a right to, it is very hard work to access the professional care and help they are entitled to. In literature, this phenomenon is described as the non-take-up of social security benefits (Van Oorschot, 1995; Ypeij & Engbersen, 2002). Van Oorschot (1995, p. xi) defines non-take-up as follows: “the phenomenon whereby people or households do not receive the amount of benefit to which they are legally entitled”. Our respondents describe their struggle as follows: one must be assertive and stand up for oneself to get one’s rights. One must have knowledge of the (ever-changing) complicated laws and regulations. One must be perseverant to get one’s rights. One must be able to anticipate one’s (progressive) disease. And one must endure being misunderstood, patronized, and stigmatized. Non-take-up does not seem to be due to the way respondents handle it.

What seems to lack in the interaction at the political-societal level is the norm of self-determination, in order to be self-reliant. According to respondents they are not the ones in the interaction who determine what is necessary and needed, but the professionals. Respondents feel that their self-determination is not taken seriously and therefore impedes their self-reliance.

This is the beginning of the paradox: the people one depends on and who should help one to become self-reliant and to participate in society, so that one can live up to the social political standard are the ones who obstruct it. In the interaction with others at the political-societal level it is difficult to get the support one is entitled to, which keeps one in the group of vulnerable people. However, a certain degree of dependency, which legally allows an appeal to government support, should be an integral part of the social political standard. Being vulnerable and not being able to be fully self-reliant due to mental illness or physical defects should be considered “normal”, and the necessary and rightful professional help and care enabling people to meet the social political standard should not be an “exclusivity” to be fought for. Our respondents have proved that they do fight for their rights and to be seen as a full and valuable member of society. This can certainly be considered a strength in vulnerability. Moreover, we discovered strength at the individual level, expressed by our respondents in terms of positive self-image and a variety of competencies. Acknowledging strength in vulnerability would allow more room and respect for the self-determination of vulnerable people.

Given the above, respondents feel that they must fully adapt. This seems to be one-way traffic moving from the individual to society. In two-way traffic, both sides need to adapt: society, social policy and those who implement and execute it on the one hand, and people from vulnerable populations and their social environment on the other. In such two-way traffic, social policy and social policy implementers should be playing a facilitating and empowering role, instead of a hindering role, in order to enable the so-called “vulnerable people” to meet the prevailing social political standards of self-reliance and social participation. Moreover, in this two-way traffic, social political standards and concepts are not only determined by an outsider’s perspective, but they also include the insider’s perspective; the perspective of those to whom the standard and concepts apply. After all, the way in which standards and concepts are interpreted determines how social services and interventions are defined and implemented.

An enabling social policy and society for so-called “vulnerable people”, in which both vulnerable and non-vulnerable people are respected, is to be recommended. This may be done by acknowledging the insider’s perspective we have presented here, and by combining this perspective with the outsider’s perspective on “vulnerable people”. Provided both perspectives are given even weight, a more complete view will emerge.

With regard to methodological issues, the selected methodology and sampling technique imply that our findings are not representative for all vulnerable people. Our findings are based on a small sample and included people from vulnerable populations known by representatives of social work organizations, and willing to participate in the study. Furthermore, our research population was selected based on selection criteria and concerned people who were able to reflect and to express themselves verbally. Qualitative research, however, never pretends that it can be generalized to a whole population. Qualitative research learns by means of particular and in-depth experiences.

Our findings expand knowledge of the concept of societal vulnerability by giving a bottom up and in-depth picture of how vulnerability is perceived by people from vulnerable populations. This perspective is not fully acknowledged in social policy and literature about allegedly vulnerable people. This study gives voice to those who otherwise remain silent. The perception of allegedly vulnerable people themselves took central place because they, as expert through experience, know what it means to be vulnerable and to be classified as a “vulnerable person”. During the in-depth interviews a number of important aspects concerning vulnerability were encountered. This is relevant for scientific knowledge building.

The respondents were very open and willing to reflect and articulate how they dealt with vulnerability in their daily lives. They expressed (both to the interviewers and the recruiting persons) that the interviews were very pleasant and that they were pleased that they were really listened to. This indicates that the interviews were also valuable to the respondents.

In our study we aimed at maximal variety and therefore included persons from different age groups, sexes, and with different illnesses and defects. Unfortunately we were not able to include allegedly vulnerable people from different ethnical and cultural backgrounds in our study. All respondents were white people with Dutch nationality and background. Further research involving interviews with allegedly vulnerable people with different backgrounds is needed to investigate the influence of ethnical and cultural diversity on perceived vulnerability.

In addition, our respondent group consisted of people with mainly physical disability, chronic psychological problems and psychosocial problems. In order to explore the influence of other (main) problems on perceived vulnerability, for example, being excluded due to sexual orientation or being involved in domestic violence, further research involving interviews with allegedly vulnerable people from other groups of vulnerable people is needed.

A limitation of our study is that we did not include other stakeholders, such as non-vulnerable people, social policy makers and practitioners who work with and/or are in touch with people from vulnerable populations, to explore their perceptions on the concept of vulnerability. In order to gain more insight into the interactional level of vulnerability, we recommend that future work should include interviews with these stakeholders.

Finally we hope that with our findings we can pave the way for more two-way traffic; for the mutual adaptation of different perspectives (insiders and outsiders). When this becomes the case, we recommend to research what the consequences would be for people from vulnerable populations.
